# Heterozygous mutations in *SOX2* may cause idiopathic hypogonadotropic hypogonadism via dominant-negative mechanisms

**DOI:** 10.1172/jci.insight.164324

**Published:** 2023-02-08

**Authors:** Jessica Cassin, Maria I. Stamou, Kimberly W. Keefe, Kaitlin E. Sung, Celine C. Bojo, Karen J. Tonsfeldt, Rebecca A. Rojas, Vanessa Ferreira Lopes, Lacey Plummer, Kathryn B. Salnikov, David L. Keefe, Metin Ozata, Myron Genel, Neoklis A. Georgopoulos, Janet E. Hall, William F. Crowley, Stephanie B. Seminara, Pamela L. Mellon, Ravikumar Balasubramanian

**Affiliations:** 1 Department of Obstetrics, Gynecology, and Reproductive Sciences; Center for Reproductive Science and Medicine; and; 2 Center for Circadian Biology, University of California, San Diego, La Jolla, California, USA.; 3 Massachusetts General Hospital Harvard Center for Reproductive Medicine and Reproductive Endocrine Unit, Massachusetts General Hospital, Boston, Massachusetts, USA.; 4 Center for Infertility and Reproductive Surgery, Brigham and Women’s Hospital, Boston, Massachusetts, USA.; 5 Gulhane School of Medicine, Ankara, Turkey.; 6 Section of Pediatric Endocrinology, Department of Pediatrics, Yale University School of Medicine, New Haven, Connecticut, USA.; 7 Division of Endocrinology, Department of Medicine, University of Patras Medical School, Patras, Greece.; 8 National Institute of Environmental Health Sciences, Durham, North Carolina, USA.; 9 Endocrine Unit, Department of Medicine, and Center for Genomic Medicine, Massachusetts General Hospital, Boston, Massachusetts, USA.

**Keywords:** Endocrinology, Neuroscience, Neuroendocrine regulation

## Abstract

Pathogenic SRY-box transcription factor 2 (*SOX2*) variants typically cause severe ocular defects within a *SOX2* disorder spectrum that includes hypogonadotropic hypogonadism. We examined exome-sequencing data from a large, well-phenotyped cohort of patients with idiopathic hypogonadotropic hypogonadism (IHH) for pathogenic *SOX2* variants to investigate the underlying pathogenic *SOX2* spectrum and its associated phenotypes. We identified 8 IHH individuals harboring heterozygous pathogenic *SOX2* variants with variable ocular phenotypes. These variant proteins were tested in vitro to determine whether a causal relationship between IHH and *SOX2* exists. We found that *Sox*2 was highly expressed in the hypothalamus of adult mice and colocalized with kisspeptin 1 (KISS1) expression in the anteroventral periventricular nucleus of adult female mice. In vitro, shRNA suppression of mouse SOX2 protein in Kiss-expressing cell lines increased the levels of human kisspeptin luciferase (hKiss-luc) transcription, while *SOX2* overexpression repressed hKiss-luc transcription. Further, 4 of the identified *SOX2* variants prevented this SOX2*-*mediated repression of hKiss-luc. Together, these data suggest that pathogenic *SOX2* variants contribute to both anosmic and normosmic forms of IHH, attesting to hypothalamic defects in the *SOX2* disorder spectrum. Our study describes potentially novel mechanisms contributing to *SOX2*-related disease and highlights the necessity of *SOX2* screening in IHH genetic evaluation irrespective of associated ocular defects.

## Introduction

Pathogenic mutations in the *SOX2* gene (encoding the SRY-related HMG-box 2 transcription factor protein) were initially identified as a monogenic cause for anophthalmia/microphthalmia (A/M) (1). Over the last 2 decades, it has become apparent that the phenotypic spectrum relating to *SOX2* mutations extends beyond A/M (2) to encompass a spectrum of other ocular defects (coloboma, optic nerve hypoplasia, hypertelorism, short palpebral fissure) and even broader extraocular phenotypes, such as neurocognitive delay, intellectual disability, brain anomalies, seizures, pituitary dysfunction, hypogonadotropic hypogonadism, genital anomalies, sensorineural hearing loss, and esophageal atresia (1). This expanded spectrum has led to the suggestion that this syndrome be referred to as a *SOX2*-associated disorder (1). Thus far, most patients with pathogenic *SOX2* variants have been ascertained through severe ocular phenotypes. Although the majority of individuals with severe ocular phenotypes harbor *SOX2* protein-truncating variants (PTVs), there appears to be an underlying allelic heterogeneity, with *SOX2* missense variants linked to milder ocular phenotypes (3). This allelic heterogeneity hypothesis requires examination in larger cohorts of patients with *SOX2* variants, especially ones ascertained by nonocular *SOX2*-related phenotypes. To date such studies have not been reported.

Among the reported *SOX2*-related phenotypes, hypogonadotropic hypogonadism (HH) represents a commonly reported nonocular phenotype (2). However, the pathogenic mechanism(s) underlying HH in these patients remains to be fully elucidated. In humans, HH can result from a deficit of hypothalamic kisspeptin or gonadotropin-releasing hormone (GnRH) secretion or action, from altered GnRH neuronal migration, or more rarely, from pituitary gonadotropin secretory dysfunction (4, 5). Initially, HH relating to *SOX2* haploinsufficiency was attributed to pituitary gonadotropin dysfunction. This notion is supported by the fact that several patients with *SOX2* disorder display anterior pituitary hypoplasia on magnetic resonance imaging (MRI) and concomitant growth hormone deficiency suggesting a pituitary deficit. Further, embryonic and adult *Sox2*^βgeo^ heterozygous mice display variable abnormalities in pituitary morphology (6–10), while *Sox2*-null mice are embryonic lethal (11). In an expansion of the phenotypic spectrum, recent data from both humans and mouse models suggest that HH resulting from *SOX2* variants actually results from GnRH deficiency rather than pituitary dysfunction (3, 7, 9, 12, 13). However, the latter observation requires additional validation in a larger cohort of patients with *SOX2* variants who manifest HH without overt anterior pituitary dysfunction.

To address these outstanding questions, we studied a large cohort of patients who were ascertained through a clinical diagnosis of idiopathic hypogonadotropic hypogonadism (IHH) in which a genotype-guided approach identified *SOX2* variants. Our IHH cohort comprised primarily individuals with hypothalamic GnRH deficiency as evidenced by our stringent clinical criteria for enrollment in our clinical research study, detailed clinical evaluation that excluded overt pituitary defects, and prior neuroendocrine characterization of our IHH cohort, which demonstrated GnRH level deficits in the majority of patients with IHH studied (4). Thus, this cohort allows us to define the contribution of hypothalamic/GnRH-related pathology to *SOX2*-related HH. Since all patients with IHH have undergone exome sequencing (ES) independent of the presence or absence of ocular phenotypes, this approach allowed us to (i) examine the relevance of *SOX2* screening in IHH and (ii) investigate the mechanisms by which these *SOX2* variants could affect transcriptional regulation of the gene.

After identifying this cohort of IHH patients with potentially pathogenic heterozygous *SOX2* mutations, we investigated the mechanistic link between *SOX2* and IHH through a variety of in vitro and in vivo techniques to establish a functional connection between *SOX2* and IHH. To elucidate the pathogenic mechanisms by which *SOX2* causes IHH, we examined (i) the expression of *Sox2* in hypothalamic kisspeptin-expressing (Kiss-expressing) cells in mice, (ii) the regulation of Kiss via direct binding of SOX2 to the Kiss promoter, and (iii) the functional consequences of human *SOX2* mutations on SOX2 function. These data provide evidence for a role of SOX2 in KISS-expressing hypothalamic neurons and establish a functional relationship between *SOX2* heterozygous variants and IHH.

## Results

### SOX2 variant spectrum in cases ascertained by IHH phenotype.

**Figure 1:**
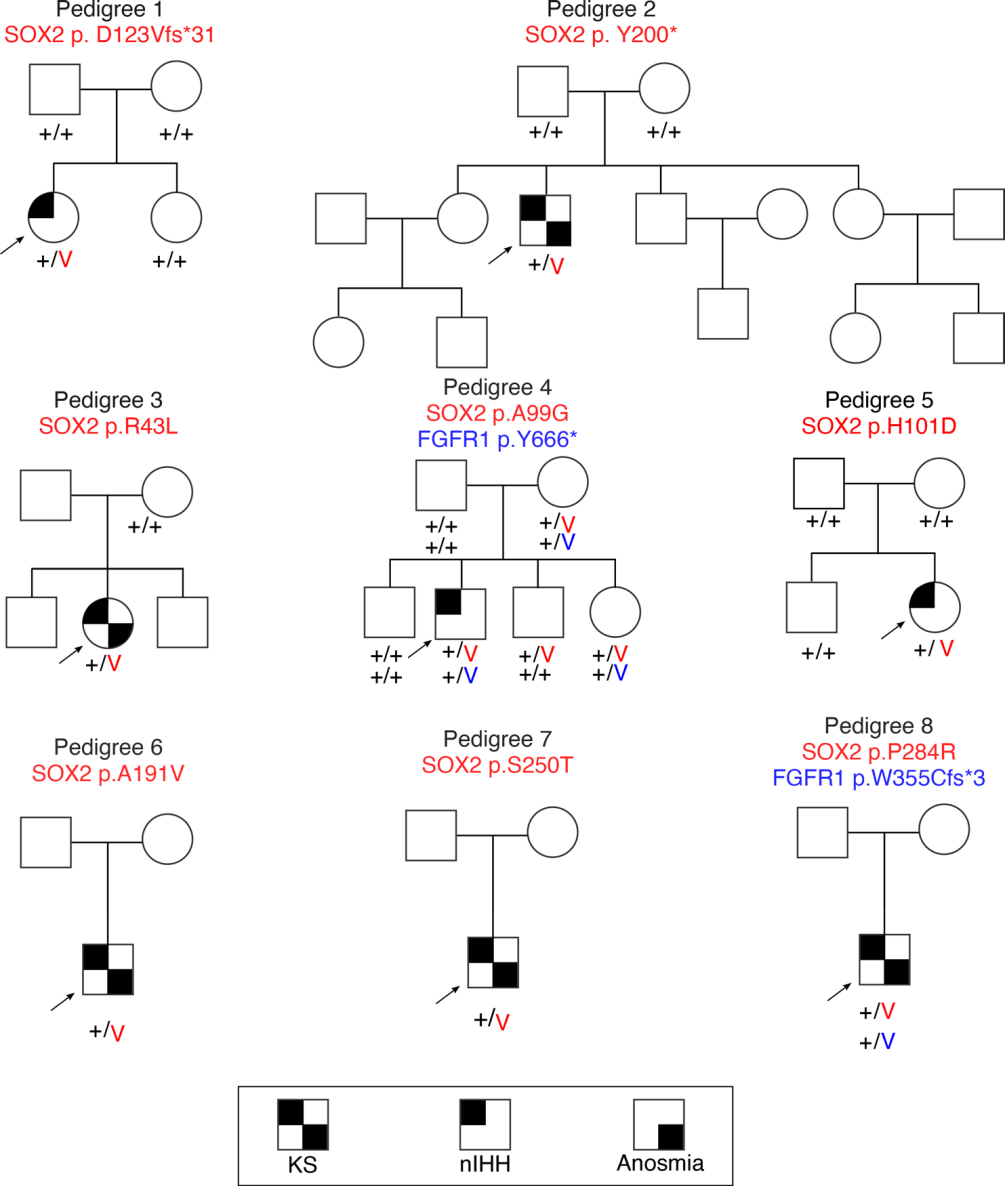
Family pedigrees of probands with *SOX2* variants identified in the Massachusetts General Hospital IHH cohort. Probands are identified by arrows; + indicates wild-type (WT) allele; and V indicates the variant allele. FGFR1, FGF receptor 1; KS, Kallmann syndrome; nIHH, normosmic IHH.

We identified a total of 8 rare heterozygous *SOX2* variants (2 PTVs; 6 missense) in the Massachusetts General Hospital Cohort, of which 7 variants (2 PTVs; 5 missense) were deemed likely pathogenic or pathogenic by applying American College of Medical Genetics (ACMG) variant pathogenicity criteria (Figure 1, Table 1, and Supplemental Table 1; supplemental material available online with this article; https://doi.org/10.1172/jci.insight.164324DS1). Six of these *SOX2* variants occurred in IHH patients without variants in other known genes while 2 *SOX2* variants cosegregated with heterozygous, protein-truncating, *FGFR1* variants (Figure 1). In silico analysis of all detected missense variants is shown in Supplemental Table 2. Overall, in our IHH cohort, 0.6% of patients harbored heterozygous *SOX2* variants, with a relatively higher number of missense alleles compared with protein-truncating alleles.

### Endocrine phenotypic characteristics in patients with IHH harboring pathogenic SOX2 variants.

The clinical characteristics of all IHH patients with *SOX2* variants are shown in Table 1. All patients failed to complete spontaneous puberty and were monitored until adulthood. Four IHH patients exhibited anosmia (i.e., the Kallmann syndromic form of IHH), pointing to joint embryonic GnRH and olfactory defects, while the other 4 were normosmic. Three male patients had evidence of neonatal hypogonadism, as manifested by microphallus and cryptorchidism. These findings pointed to severe reproductive dysfunction in most patients with *SOX2* IHH across the full reproductive life span (infancy through adulthood), except for 1 man (Table 1, case 6), who had reversal of IHH in adult life. Notably, none had clinical evidence of any other pituitary hormone deficiencies, and in cases where pituitary MRI was available, no anterior pituitary hypoplasia was noted. Taken together, these findings suggest that *SOX2* variants may lead to hypothalamic defects resulting in IHH with severe reproductive dysfunction.

### Nonendocrine phenotypic characteristics in patients with IHH harboring pathogenic SOX2 variants.

As shown in Table 1, among IHH-ascertained cases, ocular phenotypes were variable for both PTVs and missense alleles, with patients harboring *SOX2* PTVs demonstrating severe ocular defects, including right amblyopia, strabismus, blindness, and bilateral anophthalmia (Table 1, cases 1 and 2). In contrast, among all IHH patients with missense *SOX2* variants, only 1 displayed mild eye defects (myopia and small palpebral fissures) (Table 1, case 3). These observations suggest that *SOX2* variants may result in a wide spectrum of phenotypes and *SOX2* missense alleles may result in IHH with normal eyes. Thus, our findings suggest that *SOX2* screening should be considered in IHH individuals regardless of presence of ocular phenotypes.

Neurodevelopmental delay was noted in 3 patients (Table 1; cases 1, 2, and 3). Other nonocular phenotypes noted in patients included primary hypothyroidism, peripheral neuropathy, attention deficit disorder, poor fine motor skills, word processing delay, mild synkinesia, hearing loss, epilepsy, and missing molars.

### SOX2 is expressed in Kiss1 cells in adult mouse hypothalamus.

**Figure 2:**
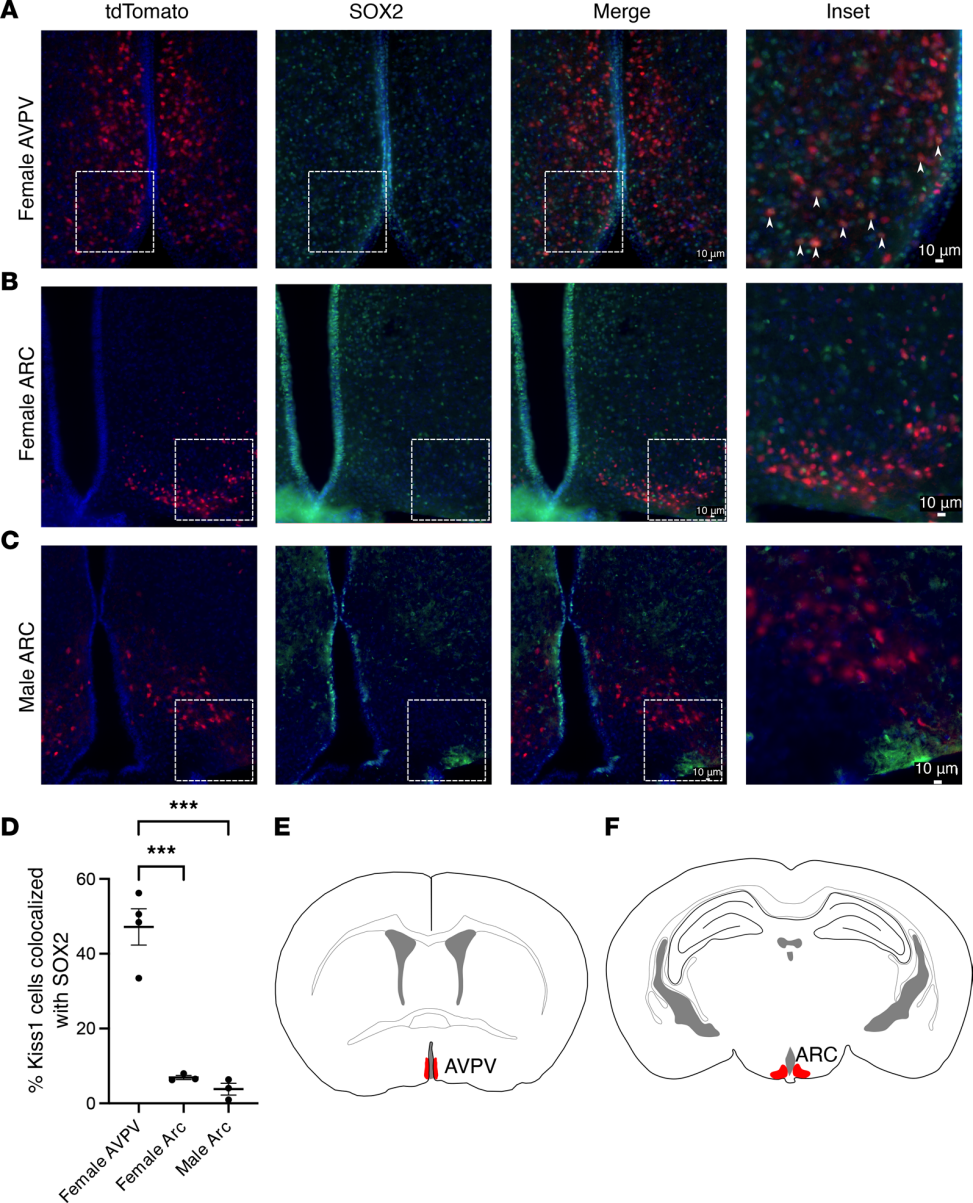
SOX2 is expressed in AVPV but not ARC kisspeptin neurons in adult mouse hypothalamus. (**A**–**C**) Kiss-tdTomato (red), α-SOX2 (green), and DAPI (blue). White arrowheads identify cells in which Kiss-tdTomato and SOX2 are colocalized. Sections are from female AVPV, female ARC, and male ARC Kiss-tdTomato reporter mice, respectively. (**D**) Quantification of the percentage of SOX2 colocalized with Kiss-tdTomato. *N* = 3 mice. A minimum of 150 kisspeptin neurons were quantified per replicate. (**E** and **F**) Schematics of the location of kisspeptin neurons in the AVPV and ARC of mice, respectively. Kisspeptin neurons were quantified throughout both regions of the hypothalamus. Schematics depict relative position of representative images in **A**–**C** with regions highlighted in red. Original image taken at 20× magnification. Scale bar = 10 μm. Data represent mean ± SEM. Data were analyzed using 1-way ANOVA with Tukey’s multiple-comparison post hoc test. Significance indicated by ***P < 0.001.

As IHH primarily results from hypothalamic defects, we sought to determine whether SOX2 was expressed in the mouse hypothalamus. A recent paper revealed that SOX2 does not colocalize with GnRH neurons (14); thus, we did not pursue the function and effect of *SOX2* in GnRH neurons further and instead focused on *Kiss1*-expressing cells. Kiss1^Cre^ mice were crossed with Rosa-tdTomato mice to create Kiss1 tdTomato mice in which all *Kiss1^Cre^-*expressing cells express tdTomato (15, 16). To visualize SOX2 expression, an anti-SOX2 (α-SOX2) antibody was used together with a fluorescently conjugated secondary antibody and counterstained with DAPI. We found that SOX2 was widely expressed in the hypothalamus of both male and female mice. Further, SOX2 colocalized with *Kiss1*-expressing cells in the anteroventral periventricular nucleus (AVPV) of adult female mice but not the arcuate nucleus (ARC) (47.16% and 6.93%, respectively. Figure 2, A, B, and D). The ARC of male mice followed the same trend, with little to no colocalization of SOX2 and *Kiss1^Cre^*-expressing cells (3.8% Figure 2, C and D). Figure 2, E, and F, provide schematics of the locations of Kiss cells in the hypothalamus. This finding suggests that *SOX2* may have a role in the function of AVPV *Kiss1-*expressing cells in the female.

### Sox2 shRNA increases the expression of hKiss-luc in Kiss neuronal cell lines.

**Figure 3:**
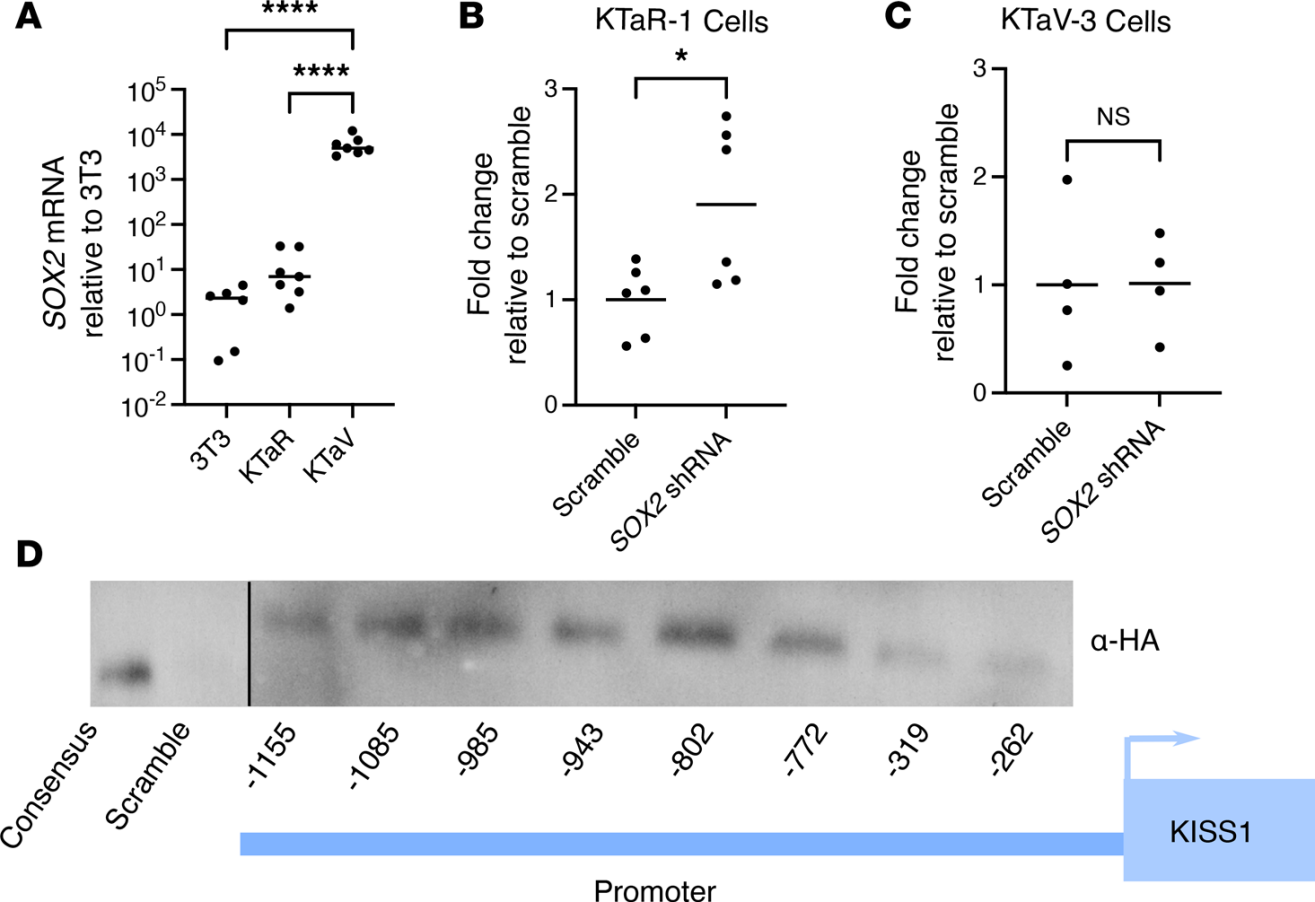
SOX2 represses KISS1 transcription in immortalized kisspeptin cell lines via direct DNA binding. (**A**) Relative mRNA levels of *Sox2* in NIH 3T3, KTaR-1, and KTaV-3 cells using real-time quantitative reverse transcription. *N* = 6–7. hKiss-luc was cotransfected with *Sox2* shRNA or a scrambled sequence in an shRNA vector into (**B**) KTaR-1 or (**C**) KTaV-3 cells. Luciferase expression in each condition was normalized to the scrambled vector. *N* = 4–6. (**D**) Biotin-labeled 30 bp double-stranded oligonucleotides from the indicated regions of the human KISS1 promoter were used in DNA pulldown of protein from NIH 3T3 cells transfected with a hSOX2 expression vector with an HA tag. Following precipitation of DNA/protein complexes with streptavidin magnetic beads, proteins were eluted and analyzed by Western blot with an α-HA antibody. The consensus oligonucleotide contains a 5× multimer of the full SOX2 binding sequence. The scrambled oligonucleotide contains a 5× multimer of a sequence unrelated to SOX2 binding. All conditions were run on same blot and with same exposure. The consensus and scramble lanes were moved to the left side for clarity with the schematic. Representative image from *N* = 3 biological replicates. Data were analyzed using Student’s *t* test or 1-way ANOVA with Dunnett’s multiple-comparison post hoc test. Significance indicated by *P < 0.05, ****P < 0.0001.

As *SOX2* is a member of the SOX family of transcription factors, we next used mouse-derived hypothalamic cell lines to determine whether SOX2 would affect the expression of *KISS1* (NCBI Gene ID: 6657). We first quantified *Sox2* expression in 2 immortalized cell lines derived from the ARC and AVPV of female mouse hypothalamus (17). These cell lines, named KTaR-1 and KTaV-3, respectively, mimic the transcriptional environment of the kisspeptin neuron and provide an in vitro model in which to conduct functional testing. As expected, given the in vivo colocalization data, we found that KTaV-3 cells contained high levels of *Sox2* RNA, while KTaR-1 cells expressed some *Sox2* mRNA but at a much lower level than the mouse fibroblast cell line, NIH 3T3, used as a control (Figure 3A). We next cloned a previously validated *Sox2* shRNA sequence utilizing a pAAV backbone and transiently transfected it into KTaR-1 and KTaV-3 cells along with a human –1,313/+27 hKiss-luc to test whether reducing the endogenous SOX2 content of the cotransfected cells would result in a change in hKiss-luc expression (18, 19). As the transfection efficiency of KTaR-1 and KTaV-3 cells is generally low, we utilized the luciferase system to allow interrogation of only the transfected cells through a cotransfected reporter as SOX2 content would only be reduced in the transfected cells. We found that *Sox2* shRNA significantly increased the expression of hKiss-luc in the KTaR-1 cells, suggesting that SOX2 functions to repress Kiss1 transcription (Figure 3B). We noticed a slight but not significant increase in the hKiss-luc expression in the KTaV-3 cells that we expect was due to the significantly higher SOX2 content in that cell line, a level that the shRNA is likely to be unable to overcome (Figure 3C).

### SOX2 binds to the SOX2 consensus sequences in the KISS1 promoter.

As *Sox2* knockdown has the capacity to increase the expression of *KISS1*, we next investigated whether this interaction might be direct via binding to the *KISS1* promoter sequences or indirect via the regulation of intermediate genes. We located putative SOX2 consensus sequences in the human KISS1 promoter. We interrogated the core region of the *KISS1* promoter included in our hKiss-luc plasmid, as previously functionally validated using Jaspar software, to identify putative SOX2 consensus motifs (https://jaspar.genereg.net/) (18). We found 8 putative binding sites. To identify which of the possible sites SOX2 functionally binds, we utilized a DNA precipitation method (20, 21). After constructing biotin-tagged, double-stranded oligonucleotides containing 30 bp sequences of each of the 8 putative binding sites, they were immobilized on streptavidin-coated magnetic beads. As all 8 of the putative binding sites were slight derivations of the full consensus sequence, both a positive control containing a multimer of 5 complete consensus sequences as well as a scrambled negative control were used. Using a custom human SOX2 expression vector (Vector Builder) modified to contain a 5′ HA tag (hSOX2), KTaR-1 cells were transiently transfected to express human SOX2 and the total lysate collected. The total lysate was washed over the immobilized oligonucleotides in binding buffer, washed several times, and then the bound proteins eluted by boiling, and the resultant protein samples were run on a Western blot using an α-HA antibody for detection. This assay found that human SOX2 binds to the consensus multimer and all SOX2 binding sites in the human *KISS1* promoter but not to the scrambled oligonucleotide (Figure 3D). This finding suggests that *SOX2* has the potential to directly regulate *KISS1*.

### Mutations in SOX2 do not alter cellular protein content of SOX2 when overexpressed.

**Figure 4:**
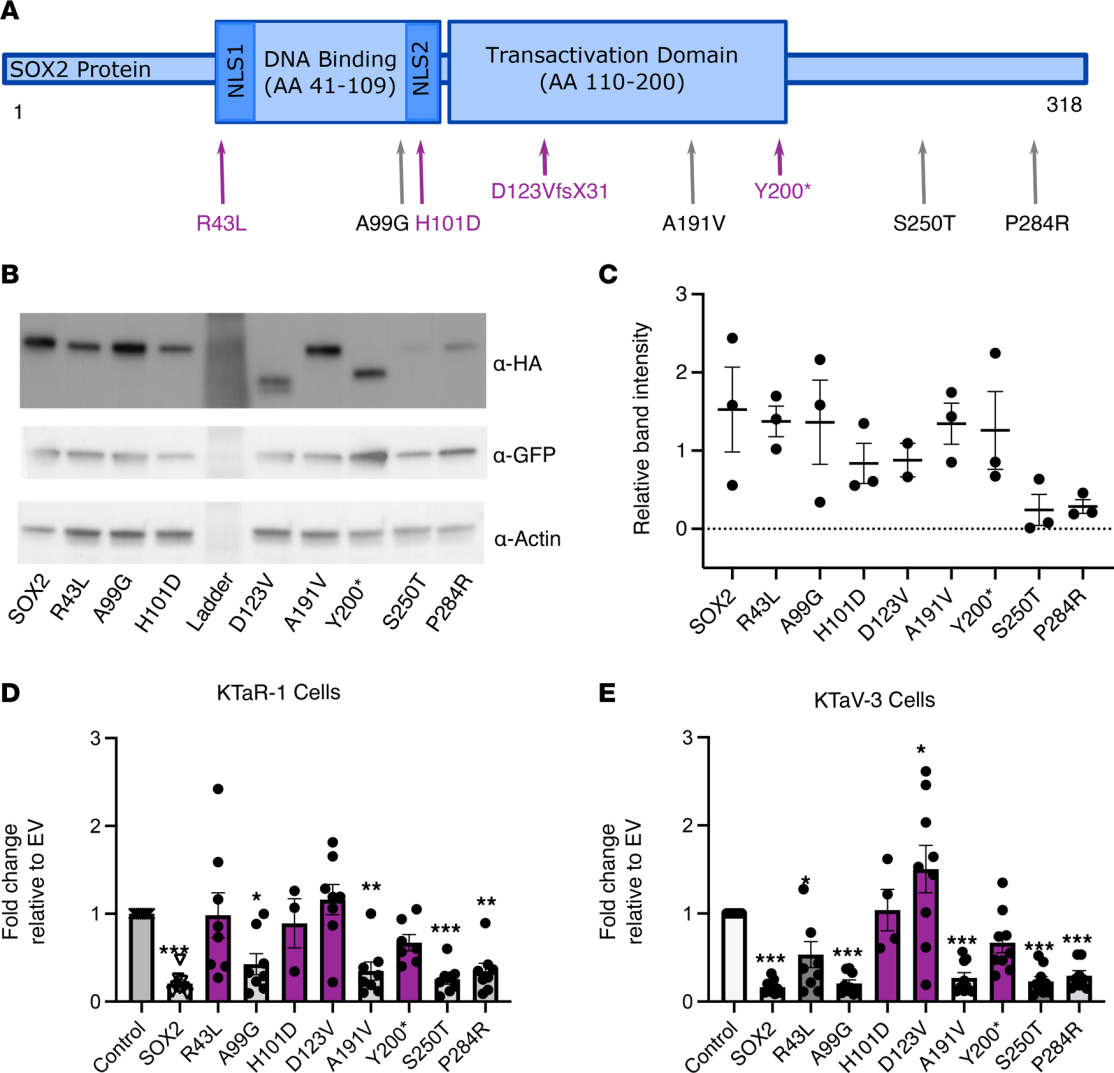
Mutations in SOX2 interfere with SOX2-mediated repression of KISS1 transcription. (**A**) Schematic of the SOX2 protein with patient mutation locations indicated. Protein mutation nomenclature follows recommendations by the Human Genome Variation Society. NLS, nuclear localization signal. (**B**) Western blot of KTaR-1 cells transfected with SOX2-HA WT or a mutation-harboring hSOX2 plasmid. (**C**) Quantification of Western blot in **B**. Total volume for each band normalized to remove background and displayed relative the transfection control, GFP. Actin used as a loading control. *N* = 3. (**D** and **E**) hKiss-luc was cotransfected with SOX2, a mutation-harboring variant of SOX2, or an empty vector (EV) into KTaR-1 or KTaV-3 cells, respectively. SOX2 protein represses expression from hKiss-luc by 79% in KTaR-1 and 84% in KTaV-3 cells. Mutant SOX2 proteins that did not repress the expression from hKiss-luc are indicated in purple in **A**, **D**, and **E**. Values were normalized relative to EV. *N* = 3–9. Data represent mean ± SEM. Data were analyzed using 1-way ANOVA with Dunnett’s multiple-comparison post hoc test if ANOVA was significant. Significance indicated by *P < 0.05, **P < 0.01, ***P < 0.001.

As described in Figure 1, we have identified 8 potentially novel variants in *SOX2* in our IHH cohort. These variants are listed using the Human Genome Variation Society nomenclature in Figure 4A, where the single-letter amino acid code is used to display the amino acid change caused by the DNA change. After finding that SOX2 is possibly involved in the transcription of *KISS1*, we sought to understand how these variants might lead to the phenotype observed in the patients. Using the hSOX2 expression plasmid with the HA tag, we created 8 new plasmids using Q5 mutagenesis, each harboring 1 mutation observed in an individual patient. These plasmids were validated by expression in our KTaR-1 and KTaV-3 cells via transient transfection followed by a Western blot using an α-HA antibody (KTaR-1 shown in Figure 4B, KTaV-3 not shown). All 8 proteins were translated into the predicted length, with the 2 mutations that lead to premature stop codons at their predicted truncated lengths (Figure 4B). As *SOX2* contains a single exon with no introns, nonsense-mediated decay is not expected to affect the protein content (Gene ID: 6657, NCBI). Further, the protein content of the cells is consistent across the mutations (Figure 4C).

### SOX2 represses KISS1 and some human variants reverse the SOX2-mediated repression.

We next used the hKiss-luc reporter plasmid and the hSOX2 plasmid to determine whether overexpression of SOX2 would affect the transcription of hKiss-luc. The WT hSOX2 plasmid and each of the 8 mutation-harboring hSOX2 plasmids described above were compared with an empty vector (EV) control, which contained the backbone of the plasmids but not the protein coding region. These plasmids were transiently transfected into KTaR-1 and KTaV-3 cells. As predicted by the knockdown experiments in which *Sox2* shRNA increases hKiss-luc expression, SOX2 overexpression represses hKiss-luc (79% repression in KTaR-1 and 84% repression in the KTaV-3, Figure 4, D and E). Further, 4 of the mutation-harboring plasmids failed to repress hKiss-luc and did not significantly differ from the EV control in the KTaR-1, KTaR-3, or both cell types (Figure 4, D and E). These mutations included 2 missense variants, p.R43L and p.H101D, and 2 PTVs, p.D123VfsX31 and p.Y200*, that introduce a premature stop codon (Figure 4, D and E). In particular, it was noted that, in the KTaV-3 cells, the p.D123VfsX31 mutation did not just reverse the SOX2-mediated repression of hKiss-luc but actually increased the expression of hKiss-luc over the levels of the EV (Figure 4E).

### Missense variants located in either nuclear localization sequence produce proteins that fail to translocate to the nucleus.

**Figure 5:**
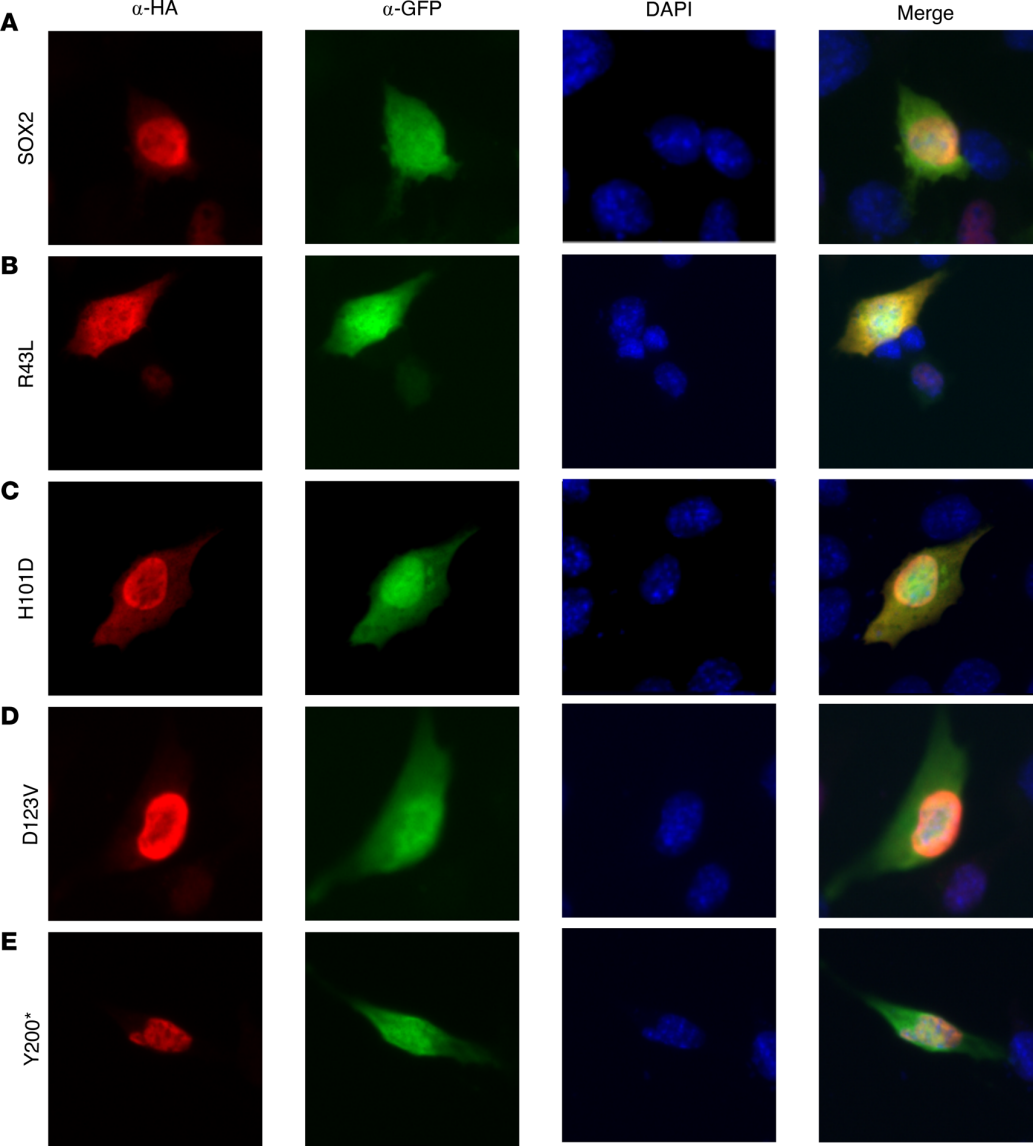
Two missense mutations in SOX2 prevent proper nuclear localization. (**A**–**E**) KTaV-3 cells were transfected with SOX2-HA WT or an hSOX2 plasmid harboring 1 of the mutations. The 4 mutations that displayed a phenotype in Figure 4, B and C, were studied. Immunostaining was completed using α-HA to identify human SOX2, α-GFP to locate transfected cells and identify the location in cytoplasm of the cell, and DAPI to identify the location of the nucleus. Representative image from *N* = 3 biological replicates. Images were taken at 40× original magnification.

Because SOX2 is a transcription factor and proper nuclear localization is required for the action of the protein on transcription, we hypothesized that the failure to repress observed in the luciferase assays for 4 of the variants might be due to a failure to translocate to the nucleus, particularly considering that the 2 missense mutations (p.R43L and p.H101D) are located in each of the 2 NLS sequences (Figure 4A) (22). We therefore transfected the WT hSOX2 and hSOX2 plasmids harboring 1 of these 4 single mutations into KTaV-3 cells and used an α-HA antibody to identify the location of the human SOX2 protein within the cell. We counterstained the nucleus with DAPI and used α-GFP, also expressed from the hSOX2 plasmid but transcribed independently of *SOX2*. GFP staining not only allows the identification of transfected cells but also fully visualizes the bounds of the cytoplasm. We found that the WT human SOX2 protein and the proteins from 2 truncating mutations (p.D123VfsX31 and p.Y200), but not the 2 missense mutations (p.R43L and p.H101D), localize correctly to the nucleus (Figure 5). This finding indicates that the 2 missense mutations may fail to repress transcription because of failure to migrate to the nucleus.

### PTVs are dominant negative and interfere with proper function of WT SOX2.

**Figure 6:**
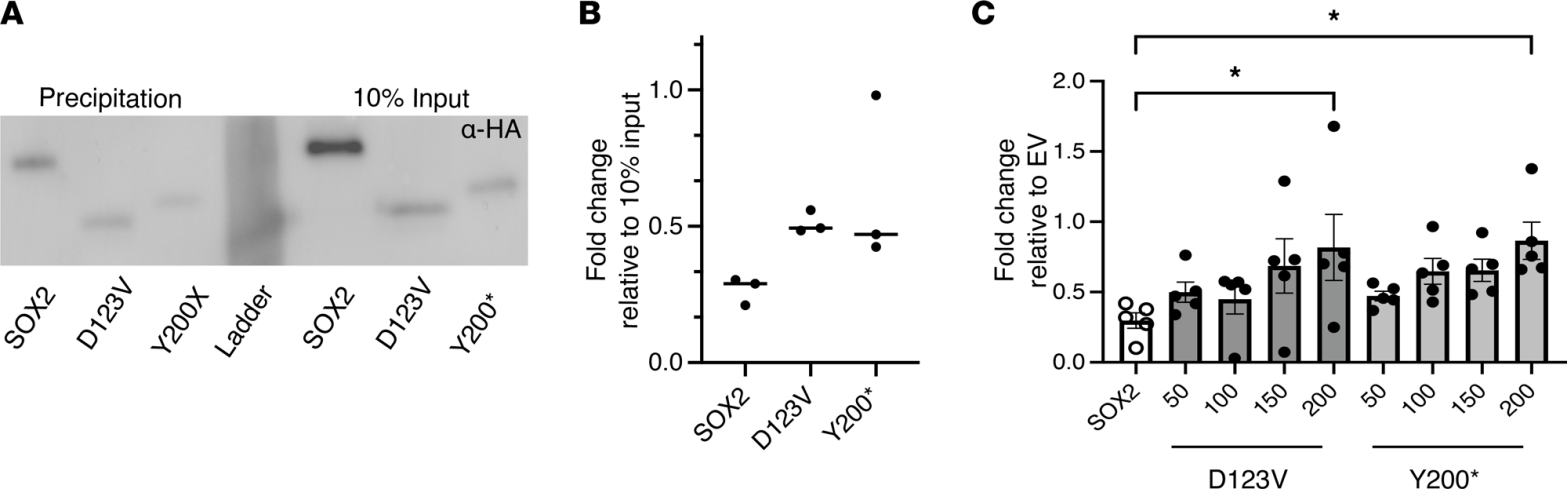
Two truncating mutations in SOX2 act in a dominant-negative fashion. (**A**) Biotin-labeled SOX2 consensus oligonucleotides were used in DNA pulldown of protein from NIH 3T3 cells transfected with hSOX2 or 1 of the mutation-harboring hSOX2 plasmids. Following precipitation of DNA/protein complexes with streptavidin magnetic beads, proteins were eluted and analyzed by Western blot with an α-HA antibody. 10% input was included as a transfection control. (**B**) Quantification of DNA precipitation in **A**. Fold-change was normalized to 10% input for each protein. *N* = 3. (**C**) hKiss-luc was cotransfected with SOX2 and a variant of SOX2 harboring 1 of the mutations in apportioned amounts from 50 ng to 200 ng. Every well also included 200 ng of WT SOX2. EV was used to compensate for differences in the amount of SOX2 harboring the mutation. Results are displayed as fold-change relative to SOX2 WT–only condition. Data represent mean ± SEM. Data were analyzed using 1-way ANOVA with Dunnett’s multiple-comparison post hoc test. Significance indicated by *P < 0.05.

Based on the results from Figure 5 and the observation from Figure 4E, in which it was found that p.D123VfsX31 increased hKiss-luc expression in the KTaV-3 cells, we hypothesized that the 2 SOX2 variant proteins with the truncating mutations might be acting in a dominant-negative fashion within the nucleus. As both mutation-harboring proteins retained their DNA-binding domain (Figure 4A), we first determined whether this DNA-binding domain remains functional. The DNA precipitation experiment shown in Figure 3 was repeated using the consensus SOX2 multimer, this time transfecting WT hSOX2, p.D123VfsX31, and p.Y200* into NIH 3T3 cells and collecting the total lysates. Western blots were performed using α-HA and normalized to 10% input. We found that the 2 truncating mutations retained their ability bind to DNA (Figure 6, A and B). Finally, the hKiss-luc assay was repeated to test for dominant-negative interference with repression by WT *SOX2*. In this experiment, 200 ng of hSOX2 plasmid was used in each condition. One condition was left as a control, containing just hSOX2 plasmid. Four conditions each of the p.D123VfsX31 and p.Y200* in a gradient from 50 ng to 200 ng of the mutation-harboring plasmids were also included. Each condition was compensated up to 400 ng total plasmid using an EV to account for differences in the quantity of DNA in each transfection. All conditions were cotransfected with the hKiss-luc reporter plasmid. The KTaR-1 cell line was used as it contained lower amounts of endogenous *Sox2* and would thus allow isolation of the effect of transfected human SOX2. We found a trend in which increasing amounts of the hSOX2 plasmid harboring the mutation increased the amount of the hKiss-luc expression, with the highest amount tested (200 ng) significantly differing from the WT hSOX2 plasmid (Figure 6C). Together, these data indicate that both the p.D123VfsX31 and p.Y200* truncated proteins retained their ability to bind DNA and interfere with the repressive function of WT SOX2 on Kiss1 transcription in a dominant-negative fashion.

## Discussion

### SOX2 is a potential cause for IHH with or without ocular defects.

Pathogenic *SOX2* variants in humans have traditionally been considered a highly penetrant cause of severe ocular defects, such as A/M (1). However, with the increasing usage of next-generation sequencing technologies and the application of genotype-first approaches, *SOX2* pathogenic variants are now recognized to result in variable phenotypic expressivity (2, 7, 12, 23–25). To ascertain whether the spectrum of heterozygous *SOX2* pathogenic variants may contribute to nonocular phenotypes, we performed *SOX2* mutational analyses in 1,453 cases ascertained by IHH, representing the largest IHH cohort surveyed for *SOX2* variants to date. This genotype-guided approach showed that *SOX2* variants (both PTV and missense) contribute to the genetic etiology of IHH. These results strongly suggest that *SOX2* screening be considered in individuals displaying any *SOX2*-associated constituent phenotype, including IHH and not restricted to patients presenting with bilateral severe ocular phenotypes.

### Heterozygous variants in SOX2 cause IHH via hypothalamic deficits rather than pituitary gonadotrope dysfunction.

A defining clinical criterion for a diagnosis of IHH in our study cohort was the demonstration of biochemical hypogonadotropic hypogonadism and exclusion of nonhypothalamic causes of HH. In addition, none of the patients reported here demonstrated any pituitary hypoplasia. Thus, this analysis points to an underlying hypothalamic deficit from either defective embryonic GnRH development or hypothalamic *KISS1* or GnRH action. To further examine the role of *SOX2* in IHH, we evaluated its expression in the mouse hypothalamus and investigated a causal link between IHH and pathogenic variants in *SOX2*. Because SOX2 is not expressed in adult mouse GnRH neurons (14), this study focuses on SOX2 function in Kiss1-expressing neurons. We have demonstrated that SOX2 is expressed in the AVPV kisspeptin neurons of adult female mice and identified a relationship between SOX2 and kisspeptin expression. Our data demonstrate that SOX2 has the capacity to repress *KISS1* directly though binding of the *KISS1* promoter region. Four of the 8 *SOX2* variants produced a protein that prevented the proper function of SOX2 regulation of *KISS1* expression. In fact, one of these variants, p.D123V, reversed the repression entirely, leading to an increase in hKiss-luc expression. We hypothesize that the expression of *SOX2* may be necessary for the proper timing and quantity of *KISS1* transcription. Several studies, both human and animal, have demonstrated that constant administration of kisspeptin leads to a subsequent decrease in circulating gonadotropins (26–28). In all cases, the pituitary is still responsive to a bolus of GnRH, which stimulates the release of the gonadotropins, just as patients with classical IHH respond to GnRH pulsatile therapy. This effect is likely mediated through the desensitization of GnRH neurons due to constitutive overexpression of kisspeptin, leading to a decrease in membrane-localized kisspeptin receptors (KISS1Rs) (29, 30). KISS1R is one of a class of G protein–coupled receptors known to be regulated through the subcellular localization of the receptor. Thus, we hypothesize that *SOX2* variants may contribute to IHH through the continued release of kisspeptin from kisspeptin neurons, leading to the desensitization of GnRH neurons to kisspeptin, a decrease in gonadotropins, and ultimately the reproductive phenotypes observed in patients with IHH.

Of note, only 4 of our 8 variants produced a phenotype in our assays. We hypothesize the lack of phenotype for some variants could be due to one of two reasons. First, a given *SOX2* variant ascertained in this study may not be the cause of the patient’s IHH. It is possible that these variants are benign in the context of IHH, despite the in silico prediction of pathogenicity, especially in those cases where patients have other cosegregating, potential disease-causing variants. This notion is supported by the finding (Figure 1, pedigree 4 [p.A99G] and pedigree 8 [p.P284R]) that 2 of the probands (p.A99G and p.P284R) also harbored heterozygous variants in the *FGFR1* gene, a well-validated IHH gene, and neither of these SOX2 proteins evidenced differences in transcriptional activity compared to WT. Second, our assays focused specifically on the role of SOX2 in kisspeptin-expressing neurons. In addition to the kisspeptin-related mechanism functionally validated in this study, we also hypothesize that SOX2 is likely acting at other points in the hypothalamic/pituitary/gonadal axis. To wit, several patients observed in our cohort had KS. This observation suggests that *SOX2* function may be involved in both olfactory and hypothalamic neurogenesis and/or migration. The association of anosmia and *SOX2* variation highlights the possibility for a role of *SOX2* in olfactory neurogenesis in humans. Indeed, in 2 previous reports of human patients with microphthalmia, 1 of whom was genetically characterized to show a 3q27 deletion spanning *SOX2*, MRI showed olfactory tract hypoplasia/aplasia consistent with disordered olfactory morphogenesis, thus lending further support to the role of *SOX2* in olfaction (31, 32). Further, prior studies have shown that while *SOX2* is not expressed in adult GnRH neurons, it is expressed in many of the supportive cells that guide GnRH neuron migration and associate with adult GnRH neurons (14). In addition, Jayakody and colleagues demonstrated a significant reduction in GnRH neuronal migration and absence of GnRH neuronal projections to the median eminence in their *Hesx1* conditional *Sox2*-knockout mice (13). Thus, it is possible that *SOX2* expression is essential for GnRH migration and function, which would further explain the relationship between *SOX2* variants and anosmic forms of IHH. To date, olfactory phenotypes have not been routinely tested or reported in patients with *SOX2* disorder, and our findings suggest that olfactory phenotyping should be incorporated in the clinical workup of these patients. Although the scope of this study did not address a causal link between SOX2 and olfactory neurogenesis, the mutation data in our patient cohort strongly suggest the need for further functional follow-up on a mechanistic link between the two. Future studies to address effects of *SOX2* mutations on the embryonic development of the hypothalamic GnRH system will be illuminating.

In addition to our data, humans with *SOX2*-related hypogonadotropic hypogonadism also showed intact pituitary responsiveness to a GnRH stimulation test (thus excluding a pituitary gonadotrope defect) and supporting a hypothalamic defect (13). Furthermore, we recently identified a patient with KS harboring a genomic rearrangement that disrupted a long noncoding RNA, *RMST*, a known binding partner for *SOX2*. This association of a key *SOX2* binding partner with the KS form of IHH lends support to its role in governing GnRH ontogeny (33). However, in addition to hypothalamic deficits, it is notable that *SOX2* variants in humans have been previously linked to several pituitary defects: anterior pituitary hypoplasia (34) and slowly progressing pituitary tumors (35). Given the well-recognized role of *SOX2* in generating major cell types in the murine pituitary gland (36), it is possible that *SOX2* disorder can result in defects at both hypothalamic and pituitary levels.

### Ocular phenotypic expressivity relating to pathogenic SOX2 alleles.

It has been previously suggested that *SOX2* missense variants result in milder ocular phenotypes compared with PTVs (3), which is supported by the results of our study. After the in vitro validation, 4 of the 8 variants were shown to be functionally impactful: 2 PTVs were dominant negative and 2 missense variants were hypomorphic. Both IHH patients with the dominant-negative PTVs demonstrated substantial eye and neurological defects. In addition, 1 of the patients with the hypomorphic missense allele of p.R43L demonstrated mild eye defects as well as mild neurodevelopmental delay, whereas the IHH patient with the de novo hypomorphic allele of p.H101D displayed healthy eyes with mild neurological defects. Those results are aligned with the notion that severe phenotypes result from haploinsufficient/dominant-negative alleles, while hypomorphic alleles can still disrupt hypothalamo/pituitary axis development without severe ocular phenotypes. Our results are also supported by prior in vitro studies in which 2 *SOX2* missense variants (p.Y110C and p.W79R) were demonstrated to be hypomorphic alleles and were associated with milder ocular phenotypes (6, 24). In addition to allelic heterogeneity, mosaicism of the *SOX2* pathogenic variant in individuals may contribute to the variable penetrance/expressivity of *SOX2*-related phenotypes (37–42). Thus, both mutational type (dominant negative and hypomorphs) and mosaicism represent key determinants of variable phenotypic expressivity in *SOX2* disorder, especially for ocular phenotypic expressivity. Indeed, genetic studies in IHH have previously shown that variants in 5 other genes (*CHD7*, *SMCHD1*, *TUBB3*, *RAB3GAP1*, and *RAB3GAP2*) result in syndromic phenotypes that include both IHH and severe ocular defects (4, 43–46). Taken together, it is likely that our current understanding of the molecular overlap between genes causing eye development and IHH is an underestimate. Future studies must evaluate pubertal phenotypes in patients with eye development disorders and vice versa.

The findings of this study have significant clinical implications. *SOX2* variants are likely to be underrecognized in patients within the *SOX2* disorder spectrum who present in nonophthalmologic settings, and such patients may not receive appropriate genetic counseling and multidisciplinary clinical care. A recent study showed that like *SOX2*, nearly 25/62 IHH genes studied had an IHH cohort prevalence of <1% (47). Despite their rarity, several of these genes are included in currently available commercial gene panels. Notably, *SOX2* is not included in most commercially available IHH genetic screening panels, and therefore, potential cases may be missed. Inclusion of all known genes for IHH (including *SOX2*) in screening panels will be important for several reasons. Defining precise genetic etiology will help avoid diagnostic odysseys for patients. Specifically, for genes like *SOX2* that result in syndromic forms of IHH, recognition of the genetic etiology will help coordinate complex case management with multiple specialists and facilitate coordinated transitions from pediatric to adult care. These subsets of patients can also be aggregated over time into larger patient registries that will further facilitate clinical translational research. In addition to missed genetic diagnosis, genetic findings in patients with IHH will have implications on disease recurrence in their offspring since fertility can be induced successfully in IHH in both sexes. Indeed, in a previous report, successful fertility induction in a *SOX2* heterozygous IHH woman without severe ocular disease resulted in a child who was born with bilateral microphthalmia (37). In this instance, the IHH individual had gonadal mosaicism for the *SOX2* variant that partly explained her attenuated phenotype. Nonetheless, variable phenotypic expressivity and the possibility of severe ocular phenotypes in the offspring must be recognized in patients with IHH pursuing fertility. For all the above reasons, we believe that inclusion of *SOX2* in gene panels and examination of *SOX2* in next-generation sequencing data sets will allow realization of precision genomic medicine for these patients.

In summary, our study findings provide robust evidence that *SOX2* pathogenic mutations impart variable penetrance and expressivity. Thus, *SOX2* screening should be considered in IHH and potentially in any patient presenting with any of the constituent *SOX2*-linked phenotypes even in the absence of severe ocular phenotypes. Findings from this study lend strong support that hypothalamic deficits (both kisspeptin and GnRH deficits) may be a primary contributor for HH phenotype in *SOX2* disorders.

## Methods

### Study patients.

The Massachusetts General Hospital IHH cohort consisted of a total of 1,453 IHH (KS and nIHH) patients enrolled in a research study within the Massachusetts General Hospital (MGH) Harvard Center for Reproductive Medicine. This cohort was clinically defined by (i) absent or incomplete puberty by age 18 years, (ii) serum testosterone < 100 ng/dL in men or estradiol < 20 pg/mL in women with low or normal levels of serum gonadotropins, (iii) otherwise normal anterior pituitary function, (iv) normal serum ferritin concentrations, and (v) normal MRI of the hypothalamic-pituitary region (4). These stringent clinical criteria allowed us to infer a hypothalamic site defect in these patients. In addition, since hypothalamic GnRH deficiency is often intertwined with olfactory dysfunction (KS form of IHH), both self-reported olfaction as well as University of Pennsylvania Smell Identification Test scores were used to classify patients as either KS (when olfaction was abnormal: anosmia/hyposmia) or nIHH (normal smell) (48, 49). For male patients who were evaluated at prepubertal age, other signs of neonatal hypogonadism were evaluated, including hypospadias, micropenis, and cryptorchidism. Clinical charts and patient questionnaires were reviewed for phenotypic evaluation for both reproductive and nonreproductive phenotypes.

The diagnosis of *SOX2* disorder was established in IHH probands in whom molecular genetic testing identified a heterozygous intragenic *SOX2* pathogenic variant, a deletion that is intragenic, or a deletion of 3q26.33 involving *SOX2* (1). Eye defects were classified as severe or mild as previously described (3). Severe eye defects were defined as bilateral ocular malformations including anophthalmia and microphthalmia. Mild eye defects were defined as unilateral anophthalmia or microphthalmia, coloboma, optic nerve hypoplasia/aplasia, strabismus, hypertelorism, nystagmus, small palpebral fissures, and myopia. Patients were evaluated for other nonocular features that have been associated with the syndrome, including neurocognitive developmental delay, seizures, hearing loss, and esophageal abnormalities, as well as genital anomalies including hypospadias, micropenis, and cryptorchidism. Patients’ pituitary function was assessed by detailed laboratory evaluation of all pituitary hormones, including thyroid-stimulating hormone, free thyroxine, prolactin, IGF1, adrenocorticotropic hormone, and morning cortisol, and pituitary anatomy was evaluated with pituitary MRIs.

### Genetic analysis.

Exome sequencing in the IHH cohort was performed on peripheral blood–derived DNA using either Nimblegen SeqCap target enrichment kit (Roche) or a custom Illumina capture kit (ICE), and the detailed variant-calling and annotation used have been described previously (50). ES data were queried for *SOX2* (RefSeq:NM_003106.4) SNVs (defined as those variants with <0.1% MAF in gnomAD, http://gnomad.broadinstitute.org) (51). We also collated variants in other genes implicated in IHH (*N* = 62) with strong human genetic evidence for causality in the literature that showed significant enrichment in gene-based burden testing in our IHH cohort (52) (Supplemental Table 2). All variants emerging from ES were confirmed by Sanger sequencing and segregation analysis performed within pedigrees for determining mode of inheritance whenever possible.

### Functional annotation of SOX2 variants.

In silico prediction of the likely functional effects of *SOX2* variants was performed using web-based software programs to analyze missense and splice site variants: PolyPhen-2 (http://genetics.bwh.harvard.edu/pph2/) (53), Sorting Intolerant from Tolerant (SIFT) (54), Combined Annotation Dependent Depletion (CADD) (https://cadd.gs.washington.edu) (55), and REVEL (56). Of note, when using the CADD program, a cutoff of 30 was utilized as scores >30 were predicted to be the 0.1% of most deleterious possible substitutions in the human genome. All SNVs were categorized using ACMG Standard and Guidelines for interpretation of sequence variants (57).

### Animals.

We crossed Kiss1^Cre^ mice (RRID: IMSR_JAX:023426; The Jackson Laboratory) (16) with Ai14 Rosa-tdTomato mice (RRID: IMSR_JAX:007914; The Jackson Laboratory) (15) to generate Kiss1 tdTomato mice, which were used as a reporter for *KISS1*-expressing cells. All mice were on a C57BL/6 background backcrossed for at least 10 generations. Adult male and female mice between 10 and 16 weeks of age were used. Mice were group-housed and maintained on a 12-hour light/12-hour dark cycle with ad libitum chow and water. Genotyping of the mice was performed by PCR with genomic DNA collected from tail tips. Cre-For: 5′-GCATTACCGGTCGTAGCAACGAGTG-3′ and Cre-Rev: 5′-GAACGCTAGAGCCTGTTTTGCACGTTC-3′ primers were used to detect the Cre allele. tdTomato was detected using tdtF1: 5′-GGCATTAAAGCAGCGTATCC-3′, tdtR1 5′-CTGTTCCTGTACGGCATGG-3′, tdtF2: 5′-CCGAAAATCTGTGGGAAGTC-3′, and tdtR2: 5′-AAGGGAGCTGCAGTGGAGTA-3′ (58).

### Plasmids.

The −1,313/+26 human KISS1-pGL2-luciferase plasmid (hKiss-luc) was provided by Alejandro Lomniczi and Sergio Ojeda (Oregon Health Sciences University, Portland, Oregon, USA) (18). The reporter plasmid containing β-galactosidase constitutively driven by the Herpes virus thymidine kinase promoter (TK-βgal) was used to control for transfection efficiency. The hSOX2 plasmid was a custom design from Vector Builder and contained the full-length human *SOX2* gene (NM_003106.4) with a 5′ HA tag under the control of an EFS promoter. The plasmids also contained GFP under the control of a CMV promoter. The plasmid was edited using Q5 mutagenesis (New England Biolabs) to produce each of the mutation-harboring plasmids. Primers used for mutagenesis are listed in Supplemental Table 3. A CMV-GFP AAV control plasmid (EV) was obtained from Addgene (plasmid 85451) and used as a control in the *SOX2* shRNA experiments. For the shRNA expression vector, the control plasmid was edited to insert a previously validated shRNA directed against *Sox2* (sense: 5′-CGAGATAAACATGGCAATCAA-3′) (19).

### Immunofluorescence.

Adult mice 10–16 weeks of age were sacrificed via perfusion. Brains were collected and fixed overnight in 4% paraformaldehyde (PFA) at 4°C followed by 2 nights in 30% sucrose at 4°C. A cryostat (Leica cm1950) was used to section the brain into 40 μm sections. Sections were blocked in PBST (PBS with 0.025% Triton X-100 and 5% normal goat serum) for 30 minutes at room temperature and incubated overnight in α-SOX2 1:1,000 (Abcam, ab97959) at 4°C. Visualization was performed using goat α-rabbit conjugated to Alexa Fluor 488 (Invitrogen) for 1 hour at room temperature. Slices were mounted onto glass slides using ProLong Gold with DAPI (Invitrogen). Quantification was performed on *n* = 3 mice per sex using 2–3 slides per mouse as needed to reach a minimum threshold of 150 cells per biological replicate. KTaV-3 cells were plated at 7,500 cells/well on 8-well Lab-Tek II Chamber Slides (Nunc) and transiently transfected with plasmids the following day via Lipofectamine 3000 reagent (Thermo Fisher Scientific) following manufacturer’s instructions. Two days after transfection cells were fixed in 4% PFA for 10 minutes at room temperature. Cells were blocked in PBST for 30 minutes at room temperature and incubated overnight in α-HA 1:1,000 (Cell Signaling Technology) and α-GFP-488 1:1,000 (Invitrogen). Visualization was performed using goat α-rabbit conjugated to Alexa Fluor 594 (Invitrogen). Wells were removed and slides were mounted using ProLong Gold with DAPI. Imaging was performed as described below. All antibodies are listed in Supplemental Table 4.

### Fluorescence microscopy and analysis.

Fluorescence microscopy for the animal tissue was performed at the Nikon Imaging Core (UCSD) using a Nikon Eclipse Ti2-E microscope with Plan Apo objectives. Samples were excited by the Lumencor SpectraX and acquired with a DS-Qi2 CMOS camera using NIS-Elements software (Nikon) acquired with an iXon Ultra 897 EMCCD camera (Andor). All slides were imaged under the same conditions. The number of tdTomato^+^ cells that colocalized with SOX2 was determined manually, using FIJI Cell Counter tool. Fluorescence microscopy for the in vitro work was performed at the UCSD Neuroscience Microscopy Imaging Core with a Keyance BZ-X Series all-in-one fluorescence microscope using Keyance software.

### Tissue culture.

KTaR-1 (RRID: CVCL_VS93) and KTaV-3 (RRID: CVCL_VS94) immortalized kisspeptin cell lines were provided by Patrick Chappell (Oregon State University, Corvallis, Oregon, USA) (17). NIH 3T3 cells (RRID: CVCL_0594) from American Type Culture Collection were used. Cells were maintained in complete media consisting of DMEM (Corning) with 10% FBS (Omega Scientific) and 1% penicillin-streptomycin (HyClone) and incubated at 37°C with 5% CO_2_. Cells were discarded after 25 passages and a fresh vial was thawed. In all experiments involving tissue culture, we counted a new passage number as a new biological replicate. Each plot represents the average of 3 technical replicates from an independent experiment performed with a different passage number.

### Luciferase assay.

KTaR-1 or KTaV-3 cells were seeded into 12-well plates (Nunc) at 20,000 cells per well. Transient transfections were performed using PolyJet (SignaGen Laboratories), following manufacturer’s recommendations. For the *SOX2* shRNA experiments, 400 ng shRNA or EV were cotransfected with 400 ng/well hKiss-luc reporter plasmids and 200 ng/well thymidine kinase–β-galactosidase reporter plasmid. For the assay in which the mutation-harboring plasmids were analyzed, cells were cotransfected with 400 ng/well expression vector, hSOX2, EV, or mutation-harboring hSOX2 plasmids, as well as 400 ng/well hKiss-luc and 200 ng/well thymidine kinase–β-galactosidase. For the truncating mutation luciferase assay, all conditions received 200 ng/well of WT hSOX2, 400 ng/well hKiss-luc, and 200 ng/well thymidine kinase–β-galactosidase. In addition, the wells received a gradient from 50 ng to 200 ng mutation-harboring plasmid as indicated in Figure 6C. All wells were compensated up to 400 ng total expression vector plasmid using the EV. Cells were plated on day 1, cells were transfected on day 2, medium was changed to stop the transfection reaction on day 3, and cells were harvested for luciferase assay on day 4 as previously described (59). Data were normalized first as luciferase activity over β-galactosidase activity and then to the control as indicated in each figure.

### Western blot.

Cells were plated on 10 cm cell culture plates (Nunc) and incubated until they reached 70% confluence. Transient transfections were performed using Lipofectamine 3000 reagent (Thermo Fisher Scientific) following manufacturer’s instructions. Two days after transfection, whole lysate was collected in 1 mL RIPA buffer (Thermo Fisher Scientific) following manufacturer’s instructions. Then 30 μg total protein lysate was run on a polyacrylamide gel 4%–20% (Bio-Rad) diluted in 1× Laemmli buffer with 200 mM dithiothreitol. Blots were transferred to a polyvinylidene fluoride membrane, blocked for 10 minutes at room temperature in TBS Blocking Buffer (Thermo Fisher Scientific), and incubated overnight in primary antibody at the following concentrations: α-HA 1:500 (Cell Signaling Technology), α-GFP 1:1,000 (Thermo Fisher Scientific), and α-Actin–HRP 1:50,000 (Abcam). Visualization was performed with goat α-rabbit IgG–horseradish peroxidase (Cell Signaling Technology) at 1:5,000 for α-HA and 1:20,000 for α-GFP for 1 hour. Imaging was done with Syngene PXi chemiluminescent detector. Blots were stripped between antibodies using Restore Western Blot Stripping Buffer (Thermo Fisher Scientific), reblocked, and reprobed for the subsequent antibody. Quantification of protein content was performed as follows. To assess SOX2 content, we probed with an α-HA antibody. To account for variation in transfection efficiency, we probed with an α-GFP antibody and normalized α-HA to α-GFP. Finally, to verify that the total protein content loaded onto the gel did not differ across samples, we verified protein loading using an α-Actin antibody. For all band intensity calculations, we used the automated Syngene PXi software as follows. The antibody band was identified in each lane, and the intensity was measured. We computed the total band intensity across all bands and calculated the individual band intensity relative to the total to account for blot-to-blot variation. All blots were performed a minimum of *n* = 3 times using independent biological replicates.

### DNA precipitation.

DNA precipitation was performed as previously described (20, 21, 59). Cells were plated and transfected as described for Western blot. Protein was collected in 1 mL FLAG buffer: 300 mM NaCl, 20 mM Tris (pH 7.5), 1% Triton X-100, 1 mM PMSF (Active Motif), 1× protease inhibitor (MilliporeSigma). Lysates were briefly vortexed, incubated on ice for 15 minutes, and spun at 15,000*g* for 15 minutes at 4°C before collecting supernatant. Then 30 μL streptavidin-coated magnetic beads (Promega) were washed twice in 2× B&W buffer (10 mM Tris pH 7.5, 1 mM EDTA, 2 M NaCl) and incubated with 100 ng/sample biotin-conjugated DNA oligonucleotides for 15 minutes at room temperature in 1× binding buffer: 5% glycerol, 20 mM Tris (pH 7.5), 1 mM EDTA, 1 mM dithiothreitol, 0.15% Triton X-100, 100 mM NaCl, 4 mM MgCl. DNA oligonucleotide sequences are listed in Supplemental Table 5. Beads were washed once in 2× B&W buffer, washed once in 1× binding buffer, and then blocked in 1× binding buffer with 1% BSA for 30 minutes at room temperature. Beads were resuspended in 50 μL of 1× binding buffer and incubated for 1 hour at 4°C with cell lysate in the following 500 μL reaction: 100 μL lysate (in FLAG buffer), 150 μL 3× binding buffer, 10 μg poly(dI/dC) (MilliporeSigma), and 50 μL beads. Beads were washed 5 times in 1× binding buffer and eluted by boiling for 5 minutes in 40 μL 1× Laemmli buffer with 200 mM dithiothreitol. Then 30 μL/sample were run as described above for Western blot. The following antibodies were used: α-HA 1:500 (Cell Signaling Technology) and goat α-rabbit IgG–horseradish peroxidase 1:5,000 (Cell Signaling Technology).

### Quantitative PCR analysis.

Cells were plated onto 10 cm cell culture plates (Nunc) in complete media and collected in TRIzol (Invitrogen) at 80% confluence. RNA was extracted using RNA clean and concentrator (Zymo) and converted to cDNA using iScript cDNA synthesis (Bio-Rad). Quantitative PCR (qPCR) was performed with the primers listed in Supplemental Table 6. We used UCSC’s in silico PCR tool to verify that our primers did not bind unintended targets. In the mouse genome, SOX2 is situated in the intron of another gene, *SOX2OT*. To ensure we amplified only *SOX2* with our primers, we used cDNA that had been processed by the cell to remove all introns. We performed gel electrophoresis of the product of standard PCR using the qPCR primers, identified only 1 product of the predicted size, and confirmed only 1 peak was present on the qPCR melt curve for all primers used. Finally, we submitted the PCR product for sequencing and validated that the product was the expected sequence. cDNA was measured using iQ SYBR Green Supermix (Bio-Rad) in a CFX Connect Detection System (Bio-Rad). A dissociation curve was performed following PCR to ensure the presence of a single product. Three technical replicates were included per experiment.

### Data availability.

Data and materials will be made available by the authors individually upon request subject to the data sharing plan and consent provided by the study participants.

### Statistics.

Gene burden testing for validating causality for the IHH genes was performed using Fisher’s exact test, where a *P* value of less than 0.05 was considered as significant. For functional analysis, differences between groups were detected by Student’s 2-tailed *t* test or 1-way ANOVA using Prism 9 (GraphPad). For 1-way ANOVA, significant effects were evaluated by Dunnett’s multiple-comparison test. Residuals were checked for normality using Shapiro-Wilk test (*P* < 0.05). When needed, data were log-transformed for plotting. Differences between groups were detected by Student’s 2-tailed *t* test or 1-way ANOVA using Prism 9 (GraphPad). For 1-way ANOVA, significant effects were evaluated by Dunnett’s or Shapiro-Wilk test (*P* < 0.05). Results were considered significant if *P* < 0.05.

### Study approval.

Clinical studies were approved by the MGH Institutional Review Board. Written informed consent was obtained from all participants or their guardians. Research activities were approved by the Human Research Committee at the MGH. All animal procedures were approved and performed in accordance with the University of California, San Diego, Institutional Animal Care and Use Committee.

## Author contributions

RB, PLM, and WFC conceived the study; JC, MIS, KWK, RAR, VFL, LP, KES, DLK, KBS, and PLM curated data; JC, MIS, KWK, PLM, and RB performed formal analysis; RB, SBS, and PLM acquired funding; JC, CCB, KES, and KJT experimented; JC, CCB, KES, KJT, RB, SBS, WFC, MO, MG, NAG, KBS, and JEH investigated; JC, KJT, RB, LP, MIS, and PLM developed methodology; JC, LP, CCB, KES, KJT, and RAR validated data; JC, MIS, LP, and KJT visualized data; JC, MIS, RB, and KES wrote the original draft; and MIS, WFC, SBS, RB, KJT, JC, and PLM reviewed and edited the draft. The order of the 3 first authors was determined by the most recent contributions and agreed upon by consensus of all the authors.

## Supplementary Material

Supplemental data

## Figures and Tables

**Table 1:**
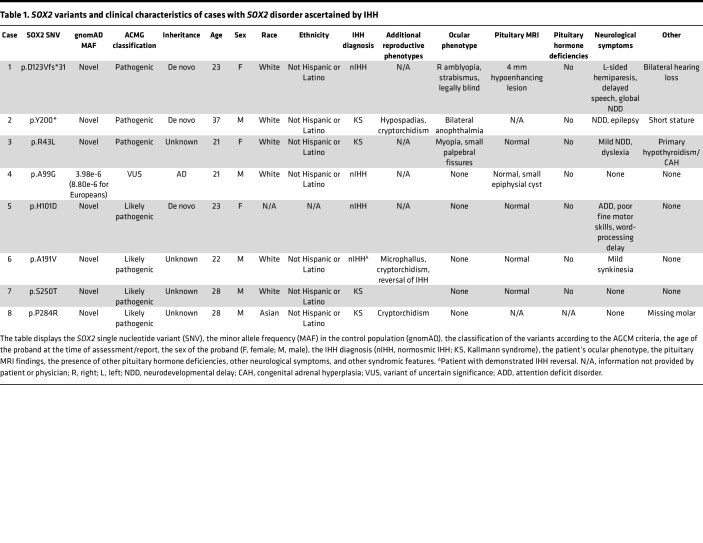
*SOX2* variants and clinical characteristics of cases with *SOX2* disorder ascertained by IHH

## Version 1 01/05/2023

In-Press Preview

## Version 2 02/08/2023

Electronic publication
