# Supplementary material for: Heterozygous mutations in SOX2 may cause idiopathic hypogonadotropic hypogonadism via dominant-negative mechanisms
Source: JCI Insight. 2023 Feb 8;8(3):e164324. doi: 10.1172/jci.insight.164324 (PMC9977424; doi:10.1172/jci.insight.164324)
Supplement: Supplemental data [file jciinsight-8-164324-s033.pdf]

Supplementary Table 1: SOX2 variants identified in IHH cases

Case	*SOX2* variant	Variant type	Inheritance	ACMG classification criteria	ACMG classification
1	p.D123Vfs*31	frameshift	*De novo*	PVS1, PS2, PM1, PM2, PP4	Pathogenic
2	p.Y200*	nonsense	*De novo*	PVS1, PS2, PM1, PM2, PP4	Pathogenic
3	p.R43L	missense	Unknown	PM1, PM2, PP2, PP3, PP4	Likely pathogenic
4	p.A99G	missense	Maternally inherited	PM1, PP2, PP3, PP4	VUS
5	p.H101D	missense	*De novo*	PS2, PM1, PM2, PP2, PP3, PP4	Pathogenic
6	p.A191V	missense	Unknown	PM1, PM2, PP2, PPP4	Likely pathogenic
7	p.S250T	missense	Unknown	PM1, PM2, PP2, PP4	Likely pathogenic
8	p.P284R	missense	Unknown	PM1, PM2, PP2, PP4	Likely pathogenic

Supplementary Table 2: *In silico* analysis of *SOX2* missense variants

Position	Change	Chromosomal location	Chromosomal location (hg19)	CADD	SIFT	PROVEAN PREDICTION	Polyphen 2
c.128G>T	p.R43L	181712488	181430276	27.4	Deleterious	Deleterious	Probably damaging
c.296C>G	p.A99G	181712656	181430444	33	Deleterious	Deleterious	Possibly damaging
c.301C>G	p.H101D	181712661	181430449	33	Deleterious	Deleterious	Probably damaging
c.572C>T	p.A191V	181712932	181430720	23.4	Tolerated	Neutral	Benign
c.748T>A	p.S250T	181713108	181430896	23.4	Tolerated	Neutral	Benign
c.851C>G	p.P284R	181713211	181430999	22.2	Tolerated	Neutral	Benign

Supplemental Table 3. Primers used for Q5 mutagenesis

Mutation	Forward primer sequence
p.R43L	5’-CGCGTCAAGCtGCCCATGAAT-3’
p.A99G	5’-CGGCTGCGAGgGCTGCACATG-3’
p.H101D	5’-GCGAGCGCTGgACATGAAGGAG-3’
p.D123V	5’-TAAGTACACGCTGCCCGG-3’
p.A191V	5’-GCGCACGGCGtAGCGCAGATG-3’
p.Y200X	5’-TGCACCGCTAaGACGTGAGCG-3’
p.S250T	5’-CGAGGCCAGCaCCAGCCCCCC-3’
p.P284R	5’-GCCGAGGTGCgGGAACCCGCC-3’

Supplemental Table 4. Antibodies

Antigen	Experiment	Concentration	Catalog number	Lot number
SOX2	IF	1:1000	ab97959	GR3386111-1
HA	IF	1:1000	C29F4	9
GFP-488	IF	1:1000	A21311	2207528
Rb-594	IF	1:1000	A11012	1933366
Rb-488	IF	1:1000	A11034	2379999
HA	Western	1:500	C29F4	9
GFP	Western	1:5000	MA5-15256	UA277185/VD299006
Actin	Western	1:50000	Ab49900	GR276781-11
Rb-HRP	Western	1:5000	7074S	30
Ms-HRP	Western	1:10000	7076S	36

Supplemental Table 5. Oligonucleotides used for DNA precipitation

Oligonucleotide Forward	Sequence
SOX2 Consensus Multimer	5’-CCTTTGTTCCTTTGTTCCTTTGTTCCTTTGTT-3’
Scramble Multimer	5’-ACGTACGTACGTACGTACGTACGTACGTACGT-3’
Kiss1 -262	5’-CCTGCCCCCAAACAACGTGGAAATGGGCTA-3’
Kiss1 -319	5’-GTGTGAAATGTCTTGTTTCCTCTCCGAAGA-3’
Kiss1 -772	5’-GGCCCCACCTTCTTGTCTCGTGGCACCCAC-3’
Kiss1 -802	5’-CCACCATACTATTTTGTGACTGCTCAGTAC-3’
Kiss1 -943	5’-CATCGCAGGTGACTTTTGTCTCTTTCTTGA-3’
Kiss1 -985	5’-ATACCCTTGGACAAGGGGCTCTCCAGGGCA-3’
Kiss1 -1085	5’-CTCTCCCAGCACAAGCTCAACCTGGTCGTC-3’
Kiss1 -1155	5’-GCACCCCTCACTTGTCTGATGCTTACTGTT-3’

Supplemental Table 6. Primers used for qPCR

Primer	Sequence
SOX2 F	5’-AAGGGTTCTTGCTGGGTTTT-3’
SOX2 R	5’-AGACCACGAAAACGGTCTTG-3’
H2AFZ F	5’-TCACCGCAGAGGTACTTGAG-3’
H2AFZ R	5’-GATGTGTGGGATGACACCA-3’
